# Behavioral Signature of Equine Gastric Discomfort? Preliminary Retrospective Clinical Observations

**DOI:** 10.3390/ani15010088

**Published:** 2025-01-03

**Authors:** Catherine Torcivia, Sue M. McDonnell

**Affiliations:** 1 Department of Clinical Studies, University of Pennsylvania School of Veterinary Medicine, New Bolton Center, Kennett Square, PA 19348, USA; torcivia@upenn.edu; 2 Havemeyer Equine Behavior Laboratory, Section of Reproduction and Behavior, Department of Clinical Studies, University of Pennsylvania School of Veterinary Medicine, New Bolton Center, Kennett Square, PA 19348, USA

**Keywords:** horse, equine gastric ulcers, EGUS, equine discomfort behavior, horse welfare

## Abstract

Gastric ulcer disease and other potentially painful gastric conditions are among the most common afflictions adversely affecting the welfare of domestic equids. A large percentage of affected animals may not display the classic signs of gastric disease, such as unexplained weight loss, poor hair coat, and inappetence until the disease becomes severe. As a clinical service within our equine referral hospital, we routinely evaluate 24-h video recorded samples of horses to assist clinicians in identifying subtle discomfort and potential sources or to scan for infrequent neurologic or cardiac-related behavioral events. Empirically, we have recognized discomfort behaviors that appear to be uniquely associated with gastric disease. These include frequent attention to the cranial abdomen (nuzzling, swatting, nipping, and/or caudal gaze focused on the abdomen caudal to the elbow) and/or deep abdominal stretching, often within the context of eating, drinking, and/or anticipating feeding. To systematically evaluate the reliability of these purported gastric discomfort behaviors, we reviewed 30 recent 24-h video behavior evaluation cases for which (1) the clinical video behavior evaluation had been carried out without knowledge of the history and presenting complaint and (2) direct gastric examination had confirmed gastric disease status at the time. Twenty-four of the thirty cases showed gastric discomfort behavior, and all twenty-four had either gastric ulcers (n = 21) and/or gastric impaction (n = 3). Of the six cases not showing gastric discomfort behaviors, four were free of gastric disease, while two had mild lesions. Comparing horses with and without gastric disease, gastric discomfort behaviors were reported in 24 of the 26 (92%) with gastric ulcers or gastric impaction, compared to none of the four gastric disease-free horses. Although a larger prospectively designed study is needed to confidently estimate the sensitivity and specificity or the associations of behavior with the type or severity of gastric disease, these results confirm our long-held clinical impression of a behavioral signature for gastric discomfort in the horse.

## Simple Summary

Gastric ulcer disease and other potentially painful conditions of the stomach are a common health concern of domestic horses, donkeys, and mules. Outward signs of gastric disease are often not readily apparent, delaying diagnosis and treatment. Based on clinical experience evaluating the behavior of horses during 24-h video recorded samples, we have recognized what appears to be an observable behavioral signature of equine gastric discomfort. Specific behaviors include frequent nuzzling, swatting, nipping, and/or gazing focused on the abdomen just behind the elbow and less commonly deep abdominal (downward dog-like) stretches, often within the context of eating, drinking, and/or anticipating feeding. To evaluate how reliably these behaviors indicate gastric disease, we reviewed clinical case records of 30 recent 24-h video behavior evaluation cases for which the stomach had also been examined. For 24 of the 30 cases, gastric discomfort behaviors had been reported, and all 24 did have either gastric ulcer disease and/or gastric impaction. Of the six cases not showing gastric discomfort behavior, four were free of gastric disease and two had mild gastric lesions. Comparing horses with and without gastric disease, gastric discomfort behaviors were reported in 24 of the 26 horses with gastric ulcers or gastric impaction, compared to none of the four disease-free horses. These findings support our long-held clinical impression that when these behaviors are seen, whether in casual observation or video evaluation, follow-up gastroscopy should be considered to definitively diagnose the horses so that appropriate treatment can be prescribed.

## 1. Introduction

Gastric ulcer disease and other potentially painful gastric conditions are among the most common afflictions adversely affecting the welfare of domestic horses, donkeys, and mules. Estimates of prevalence of gastric ulcers in horses based on gastroscopic or postmortem diagnosis vary among studies, breeds, and types of work. Nonetheless, based on recent reviews of prevalence studies among various breeds and uses, it is reasonable to expect that currently at least 50% of horses, especially in training or work, are affected [1,2]. Traditionally, the common presenting complaints leading to gastroscopic examination have included unthriftiness, picky appetite, weight loss, dull hair coat, recurrent colic, or otherwise unexplained decreased performance. However, it is becoming clear that many horses with gastric disease do not exhibit these traditional signs and so likely are not suspected of having gastric disease. More recently, it has been recognized that certain specific behavioral problems are associated with gastric disease. Our clinical experience has been that many horses that are eventually diagnosed with gastric ulcers have a history of non-specific behavior or performance problems such as reluctance to work, social incompatibility, or a sour attitude. These problems are often misattributed to inadequate training, “inadequate discipline”, poor rider skill, reproductive hormones, or other physical causes. In addition to delayed diagnosis and treatment of gastric disease, these horses are often subjected to behavior modification interventions that may further threaten their welfare. One such undesirable behavior is girth aversion, known as girthiness. This involves aggressive threats toward the handler during saddling or being groomed over the cranial abdomen. A review of 37 cases of girth aversion presented to a veterinary school referral hospital revealed that most were diagnosed with conditions involving presumed physical discomfort, including 12 with gastric ulcers [3]. Certainly, from a welfare and human safety perspective, there is a need for a better understanding of the relationship of equine gastric discomfort with problem behavior and methods for improved detection of gastric disease.

**Figure 1:**
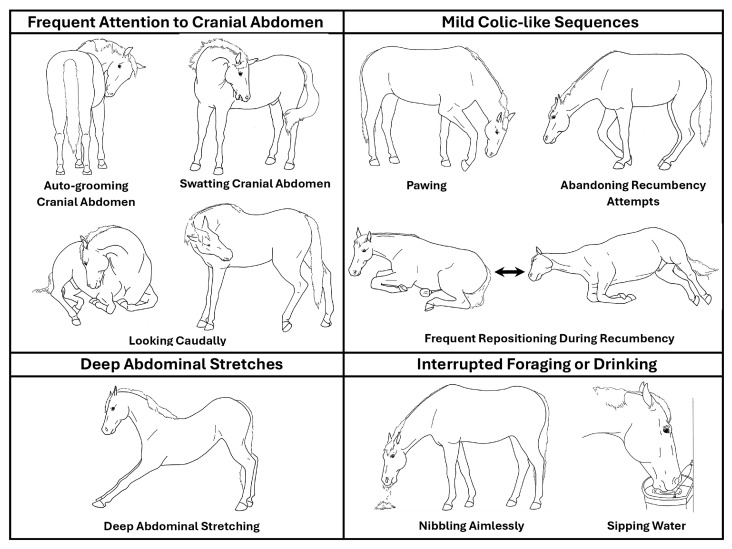
Gastric discomfort behaviors.

Our clinical equine behavior service at the University of Pennsylvania School of Veterinary Medicine New Bolton Center routinely provides behavior evaluation of 24-h video recorded samples of horses. The purpose of these evaluations is to identify any behaviors suggesting potential sources of physical or social/environmental discomfort and to capture examples of suspected infrequent events such as seizure, syncope, and sleep deprivation-type collapse [4,5,6]. In that work, we have recognized certain specific discomfort behaviors that appear to be associated with and, in certain combinations and circumstances, relatively unique to gastric disease including gastric ulcers and gastric impaction. Briefly, as shown in Figure 1, specific discomfort behaviors that we have judged to be associated with gastric disease include (1) frequent attention to the cranial abdomen including gazing, swatting, and/or auto-grooming (nuzzling, nipping) at the cranial abdomen, specifically just caudal to the elbow, usually bilaterally, (2) mild colic-like sequences of caudal gaze specifically focused on the cranial abdomen just behind the elbow, with pawing and/or circling as if intending to lie down, and/or frequently changing positions when recumbent, (3) deep abdominal (downward dog-like) stretches, and (4) interrupted or tentative foraging or drinking (picky eating and sipping water tentatively). These behaviors typically occur in clusters of various combinations, accompanied by various non-specific discomfort behaviors, such as ears focused caudally, swishing or slapping tail movements, weight shifting, hind limb lifting and/or kicking toward the abdomen, and sympathetic surge resolution signs (tongue extension, chewing, swallowing, head and neck rotational shaking, rubbing face to forelimbs). The discomfort events often occur in association with lying down or rising, upon first ingesting food or water after a period of not eating, approach/avoidance conflict behavior when eating or drinking, food urgency behavior, and/or food-related aggression toward conspecifics or caretakers. These behaviors, particularly within those contexts, can be distinguished from those seen with caudal gastrointestinal tract discomfort (e.g., classic colic). These and other equine discomfort behaviors have been detailed with example video clips in an open access equine discomfort ethogram [6].

In order to evaluate this long-held clinical impression of a relatively unique cluster of behaviors associated with gastric disease, we retrospectively reviewed clinical records of cases for which we had both 24-h video behavior evaluation findings and reports of contemporary gastroscopic or postmortem examination of the stomach to confirm whether or not gastric disease was present at the time of behavior evaluation. We considered the review of these case observations important preliminary work for subsequent prospectively designed studies.

## 2. Materials and Methods

The medical records of equine behavior consult cases of the University of Pennsylvania School of Veterinary Medicine’s hospital overseen by various clinical services during the period of January 2020 through November 2024 were reviewed to identify cases for which both (1) a 24-h video behavior evaluation had been completed by a clinician who at the time was blind to the presenting complaint, history, or planned diagnostics, and (2) information was available in the medical record concerning the gastric disease status of the patient at the time of the behavior evaluation (confirmed by gastroscopy or postmortem examination). Thirty cases meeting those selection criteria were identified. These cases had been referred to either our neurology, sports medicine and imaging, cardiology, reproduction, and/or surgery services. Presenting complaints included primarily performance problems and behavioral changes referred for neurologic, musculoskeletal, respiratory, endocrinologic, reproductive, and/or cardiac evaluation. Of the 30 cases, only 1 had been referred specifically to have gastroscopy while hospitalized primarily for other reasons. Breeds included Thoroughbred, Quarter Horse, Morgan, Arabian, Pony of the Americas, Dutch Harness Horse, Oldenberg, Holsteiner, Dutch Warmblood, Danish Warmblood, Friesian, and other crossbreeds.

For all 30 cases, our routine video behavior evaluation had been conducted by the same experienced behavior clinician (SMM). Details of our clinical evaluation method and equine discomfort ethogram are available elsewhere [4,5,6]. In brief, the evaluation included the viewing of a continuous 24-h digital video-recorded sample (GoPro Hero 4 Silver, 720p 30fps, GoPro Inc., San Mateo, CA, USA) of the horse in a stall, with care staff advised to interact and continue care and feeding as usual. Video scanning was conducted primarily in fast forward (generally 20 × real time), stopping and reviewing to clarify particular behaviors as needed. Any repetitive discomfort behaviors observed along with their apparent anatomical or system focus and context were noted. Additionally, the overall resting and foraging behavior pattern, the response to environmental stimuli, the temperament in terms of interaction with care staff, the social response to other animals, and other events of note were recorded. A narrative summary report along with screenshot and/or video examples illustrating specific discomfort behaviors was provided to clinicians and uploaded to the electronic medical record. The description of frequent discomfort behaviors was typically reported qualitatively rather than quantitatively. For each of the 30 qualifying cases, the 24-h video behavior evaluation report was retrospectively reviewed to determine whether or not observations of gastric discomfort behaviors had been described and to catalog any associated specific gastric discomfort behaviors mentioned. For each case, the medical record was reviewed for information regarding gastric disease diagnosis from either gastroscopy or postmortem examination of the stomach. The examinations had been conducted by various clinicians and full examination reports were not available for review. Accordingly, the detail and grading of gastric lesions reported in the discharge notes varied. Video behavior evaluation reports and corresponding gastric examination findings as reported in the discharge notes were summarized.

## 3. Results

Table 1 lists for each case the specific behaviors observed during the 24-h video samples and the corresponding reported gastric examination results as described in the clinician’s discharge notes. For 24 of the 30 cases, the behavior evaluation report included gastric discomfort behaviors. All 24 had gastric disease. Twenty-one had mild-to-severe gastric ulcer lesions, one had both gastric ulcer lesions and gastric impaction (postmortem examination), and the remaining two had gastric impactions that prevented gastroscopic examination of the mucosa. Specific clusters of the reported behaviors varied. Twenty-three of the twenty-four (96%) were reported to bilaterally gaze or swat at, nuzzle, and/or nip at the cranial abdomen immediately behind the elbow. In 13 of the 23 cases, this occurred within the context of returning to foraging after a period of rest, while anticipating feeding or ingesting a grain meal, and during and/or rising from recumbent rest. Eight of the twenty-four (33%) showed multiple deep abdominal stretches. Seven of the twenty-four (29%) showed both frequent bilateral attention to the cranial abdomen and deep abdominal stretching.

For the six cases for which the 24-h video behavior evaluation report did not include observations of behavior suggesting gastric discomfort, four (66%) had no stomach lesions. One of the remaining two had lesions described as “mild glandular and very mild squamous lesions”, and the other had focal hyperkeratosis.

Comparing the behavior reported for horses with and without gastric disease, 24 of 26 (92%) with disease showed frequent attention to the cranial abdomen and/or deep abdominal stretching, while none of the four disease-free horses showed those behaviors. The difference was highly significant (Fisher’s Exact Test, *p* = 0.0005).

Post-treatment follow-up that included both video behavior evaluation and gastroscopy was available for only three of the 26 horses diagnosed with gastric ulcers. For two of these three horses, the gastric ulcers had healed and gastric discomfort behavior was no longer observed. The third had been a case of gastric impaction at the time of the initial evaluation. Upon follow-up, gastric emptying was reported to have improved somewhat and discomfort behavior was reported as less frequent and animated than during the initial evaluation.

## 4. Discussion

These results support our long-held clinical impression that certain discomfort behaviors, especially within certain contexts, are consistent with gastric disease in horses. In this sample, all 24 cases that exhibited those discomfort behaviors had confirmed gastric disease. Although there were only four gastric disease-free horses in this sample, it is notable that none of those four showed gastric discomfort behavior. Only two of 26 horses with gastric lesions, both with only mild lesions, were not reported to have those behaviors in a 24-h video-recorded sample. Therefore, the absence of observation of gastric discomfort behavior in a 24-h video behavior evaluation does not necessarily indicate the absence of gastric disease. Clearly, prospective evaluation of a greater number of cases would be required to confidently estimate how reliably the absence of gastric discomfort behaviors indicates the absence of gastric disease.

For this sample, the particular combination of specific discomfort behaviors noted varied among individual horses. The most commonly reported was frequent bilateral attention to the cranial abdomen behind the elbow (gazing toward, swatting and/or nuzzling, nipping). In most cases, this occurred more frequently in anticipation of feeding, after intervals without access to feed or hay, when first ingesting hay or grain after an interval without eating, or upon lying down or rising from recumbent rest or rolling.

The behavior observations described here are all based on the review of video recordings. It has been established that ongoing discomfort/pain behaviors of horses typically cease or are greatly reduced in the presence of people compared to when alone and undisturbed [7]. The Horse Grimace Scale (HGS) is a rubric developed for the recognition of pain in horses based on systematic evaluation of the tension of various facial muscles [8]. Recently, Ferlini and co-workers [9] reported that HGS scores failed to distinguish between horses with and without gastric ulcers or among horses with mild, moderate, or severe gastric lesions. Their HGS evaluation was based on a series of photographs that had been obtained in an in-person photo session which involved considerable manipulation of the horse and disturbance of ongoing behavior. Although the effect of caretaker presence on HGS scores has not yet been critically evaluated, it is reasonable to expect that HGS scores would be similarly altered by the presence of the person obtaining the photographs. In our clinical video behavior evaluations, we have not specifically recorded HGS scores. However, it is likely that facial expressions, along with other non-specific discomfort postures and behaviors, influence our assessment of specific discomfort behaviors. It may be worth evaluating whether our clinical video recordings provide views of facial expression sufficient to address the question of whether facial expression of pain changes in the presence of caretakers or other disturbances.

While these gastric discomfort behaviors may not occur in the caretakers’ presence, the frequent nuzzling and nipping of the abdomen commonly leaves the target area conspicuously marked (wet and/or roughened haircoat; missing hairs and skin blemishes). This is something that caretakers can easily recognize during routine grooming and daily interaction. Of course, there could be alternative explanations for oral attention to the area such as saddle girth sores or other skin conditions, but especially when bilateral, and with otherwise healthy skin, the marking likely reflects gastric discomfort behavior.

In this retrospective clinical case review, there did not appear to be an obvious association of specific elements of discomfort behavior with the severity of disease or with the type and location of lesions as reported. Working from discharge notes, an obvious limitation was the variation among cases in the details of lesions described. Recently, in a model of experimentally induced gastric ulcer disease, both pawing and eating bout frequencies were found to be significantly greater in the four horses with severe (Grade 3 or 4 on a 0 to 4 scale) ulceration than in the four horses with mild (Grade 0–2) ulceration [10]. A prospective study of a larger sample of horses under more controlled conditions than in the case of our hospitalized patients would more appropriately address the associations of these other gastric discomfort behaviors with the severity of disease.

It has also been our clinical impression that when gastric disease is successfully treated, these discomfort behaviors are no longer seen. In this particular sample, follow-up video behavior evaluation and gastroscopy following treatment had been carried out for three of the 24 cases. For two of those horses, the ulcers had healed and no gastric discomfort behavior that had been noted during the initial video evaluation was noted. The third was a horse that had gastric impaction. Follow-up gastroscopy indicated less retention of ingesta. Follow-up video behavior evaluation indicated less frequent and intense gastric discomfort behavior. Although there was only a small number of cases with follow-up, these observations lend additional support for the association of these behaviors with gastric disease.

These results support the concept that these gastric discomfort behaviors, whether informally observed by caretakers or reported in formal 24-h video behavior evaluation, can increase our confidence that gastric examination should be recommended. In our clinical experience, the question often arises as to whether gastroscopic examination is necessary or whether to just proceed with treatment. In recent years, gastric ulcer disease has been recognized as two distinct types, squamous and glandular, in terms of pathophysiology, risk factors, response to treatment, and therefore recommended treatment [11]. Accordingly, direct examination of the stomach is necessary to appropriately recommend treatment. In addition, it is important to note that among the 24 cases with gastric disease reported here, 3 (12.5%) were found to have gastric impaction, for which an entirely different treatment protocol is recommended [12]. This further supports the need for direct visualization to reach specific diagnosis/diagnoses before commencing treatment.

Our standard clinical video behavior evaluation is based on fast-forward scanning of a full 24-h sample which typically requires 1–2 h to complete. Clearly, obtaining and evaluating 24-h video samples may not be a practical approach to screening for gastric discomfort. Nonetheless, many equine facilities already have 24-h video surveillance/recording systems in each stall, and many caretakers/owners routinely monitor and/or scan recorded footage. In our clinical and research experience, gastric discomfort behaviors typically occur throughout a 24-h period. Moreover, gastric discomfort events typically occur more frequently in association with feeding grain meals. We often find that just a 1- to 2-h sample before, during, and after feeding is often sufficient to detect gastric discomfort behavior. Gastric discomfort behaviors are relatively conspicuous even when viewing in fast forward up to 20 times the real speed. Therefore, typically 2h to 4 h of video can be reviewed for this purpose in less than 15 min.

The application of artificial intelligence methods (AI) for use in recognizing animal behavior is moving quickly since its recent introduction [13]. Reliable AI detection of gastric discomfort behavior could greatly improve animal welfare. Researchers have already begun exploring AI evaluation of behavioral signs of classic colic, a painful abdominal condition with a set of discomfort behaviors similar to some of the responses seen with gastric discomfort [14]. It is expected that these current results supporting the reliability of specific behavioral responses and patterns that distinguish gastric discomfort will promote the development and refinement of AI detection methods specific to each condition.

The present retrospective clinical case review has obvious limitations. Most conspicuously, very few gastric disease-free cases met our inclusion criteria. Even though none of the four disease-free horses showed the purported signature behaviors, greater numbers would be needed to make meaningful estimates of specificity. Even though none of the horses in this sample had been referred specifically for evaluation of gastric disease, our hospital population likely reflects the high end of estimates of gastric disease prevalence [1,2]. Secondly, a larger sample of horses with gastric disease would strengthen estimates of sensitivity, even for mild lesions or hyperkeratosis. Thirdly, critical evaluation of the association of the type and severity of gastric disease with behavior is limited by our dependence on clinical behavior evaluation summaries by clinicians and clinician discharge notes. Ideally, the findings reported here will be followed with prospectively designed studies that include (1) a greater number of both gastric-diseased and disease-free horses, (2) detailed, standardized descriptions and grading of gastric lesions, (3) quantitative evaluation of the frequency and intensity of these and possibly additional discomfort behaviors and their contexts, and (4) post-healing follow-up gastroscopy and behavior evaluations. Additionally, further studies comparing the discomfort behavior of horses with caudal abdominal gastrointestinal conditions or other caudal abdominal visceral conditions, both with and without gastric disease, would be helpful to confirm the distinguishing behaviors.

## 5. Conclusions

In this sample, certain discomfort behaviors, notably frequent attention to the cranial abdomen (nuzzling, swatting, nipping, and/or caudal gaze focused on the area immediately caudal to the elbow) and/or deep abdominal (downward dog-like) stretches, reliably indicated gastric disease. The only two of the 26 diseased horses that did not show these discomfort behaviors had mild lesions or hyperkeratosis. Notable, none of the four gastric disease-free horses showed those behaviors. These results support our long-held clinical impression of a behavioral signature for gastric discomfort in the horse and confirm our long-held clinical recommendation that when these behaviors are seen, whether in casual observation or clinical video evaluation, follow-up gastroscopy should be considered to definitively diagnose the horse so that appropriate treatment can be prescribed. A prospectively designed study of larger numbers of both gastric disease-free and diseased cases is needed to confidently estimate the sensitivity and specificity or the associations of behavior with the type or severity of gastric disease.

## Figures and Tables

**Table 1:** Summary of video behavior evaluation and gastric examination reports for sample of 30 equine cases.

24-h Behavior Evaluation Report					Gastric Examination Report
Case	Attention to Cranial Abdomen	Paw	Deep Abdominal Stretch	Other ^b^	
1	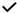 ^a^		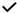		ulcers diagnosed by referring veterinarian
2	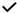 ^a^				grade 3/4 squamous lesions, mild glandular lesions
3	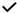 ^a^		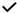		grade 2/4 squamous lesions
4	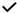			D	gastric ulcers diagnosed by referring veterinarian
5		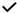	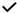	A, B, C	grade 2/4 squamous and severe glandular lesions
6	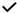 ^a^			A	few small squamous lesions, hyperkeratosis
7	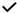 ^a^				ulcers diagnosed by referring veterinarian
8	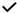				ulcers diagnosed by referring veterinarian
9	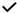 ^a^	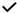	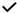	A, D, E	ulcers diagnosed by referring veterinarian
10	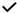 ^a^				squamous, glandular, duodenal lesions
11	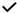		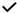		squamous, large area of glandular lesions
12	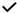 ^a^	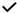	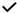		full gastric impaction, unable to view mucosa
13	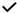				squamous and glandular lesions
14	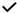 ^a^	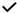	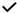	D	mild chronic squamous lesions
15	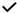	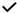		D	gastric impaction and severe squamous lesions
16	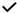 ^a^	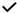		D	multifocal pyloric lesions
17	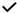	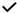		F	grade 2/4 squamous lesions, pyloric hyperemic
18	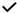			A	grade 2/4 squamous ulcers, multifocal glandular, multifocal hemorrhagic ulcers around pylorus
19	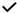		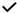	A, D	pinpoint glandular ulcers, erythema around pylorus
20	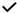	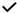		A, D	severe ulcers and plaques
21	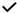 ^a^	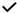		A, D, E	gastric impaction
22	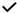 ^a^				grade 3/4 squamous ulcers
23	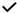			C	glandular and pyloric ulcers
24	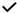 ^a^				multifocal chronic squamous ulcers
25					very mild squamous and mild glandular lesions
26					focal hyperkeratosis over the lesser curvature
27					no ulcerative lesions (some flaking mucosa)
28					no lesions
29					no lesions
30					no lesions

^a^ associated with resuming foraging after a rest, anticipating or ingesting grain meal, and/or during or upon rising from recumbent rest. ^b^ Other Behaviors: **A** interrupted, reluctant eating, appearing uncomfortable (e.g., pawing, grimace, frustration head shaking, tail swish, yawn, hyper-vigilance, hyper-reactivity); **B** sip water or interrupted drinking; **C** dip hay in water; **D** kick or draw limbs toward cranial abdomen; **E** food aggression, food guarding; **F** mild colic recumbency/intention sequence (paw, circle, lie down, interrupted recumbency).

## Data Availability

No additional data were created.
